# Mr. Custance, on Vaccination

**Published:** 1804-03-01

**Authors:** George Custance

**Affiliations:** Kidderminster


### *To the Editors of the Medical and Physical Journal.*

GENTLEMEN,

THE maxim, "Veritas est magna et prævalebit," was never more strikingly exemplified, than by that complete victory which the cow-pox has already obtained over every description of its opposers. The ignorance and indolence of some, and the selfishness and prejudice of others, were soon found to be barriers too weak to resist the mighty torrent, which, sweeping away every obstacle before it, has now inundated almost the whole civilized world; and it can scarcely be doubted, but that in time it will hurry the small-pox, with all its dreadful consequences, into the ocean of oblivion. Perhaps there are individuals amongst the more enlightened part of the community, who still resist the evidence in favour of vaccination; but scarcely will a *professional* man be found so destitute of common sense, or so regardless of his reputation, as to attempt to discredit it.

The infallibility of cow-pox, as a preservative against the small-pox, is fully established; and it is hoped, that subsequent experiments will confirm the opinion of those who already believe in its powers over the plague. And whilst we behold its virtue in preventing the accession of one or more diseases, I am not without hope that time and observation will evince its efficacy in *removing* others of an alarming and fatal tendency, and thus entitle it to the character, both of a *prophylactic* and a *therapeutic*.

I am led to this hope by the successful treatment of three cases cases of *atrophia* by vaccination within the last year. Dr. Cullen has thus defined this disease, " Marcor et asthenia, sine pyrexia hectica," and makes it the second genus of the order " Marcores," which he describes as " Corporis totius macies." The subjects of these three cases were all under six months old, and I think had that species of the disease which Dr. C. calls " Atrophia (famelicorum) a nutrimento deficiente," and Sauvage " Atrophia lactantium." The emaciation of each was advanced to a very great degree, and they who have been accustomed to see this disease in children will understand me, when I say that the countenance in each was very much like that of a young monkey. The first of these children I vaccinated, without having any other idea than that of preserving it from the small-pox; but I was so struck with the rapid amendment of its general health, immediately after the scabbing process was completed, that I inoculated the other two by way of experiment, and was happy to find the event the same in all three. They are now healthy children, two of them grown plump and fat, and the other daily advancing to the same condition. I inoculated a fourth child, under similar circumstances, but it died before the scabbing process was finished. I was fearful this event would have brought discredit upon the cow-pox, but was happy to find that the parents of the child were convinced that the inoculation had nothing to do with its death, which they were satisfied was occasioned by its former disease. I think that scrophula has much to do with this disease, and that the deficiency of nutriment arises not from a sufficiency of food not being taken into the stomach (for these children were all very hearty) but from a diseased state of the mesenteric glands, which either prevents a due quantity of aliment from passing into the thoracic duct, or by changing the properties of the chyle, renders it unfit for the nourishment of the body. " But how," it may be asked, " can the cow-pox remove a diseased state of the glands?" I answer, " How can the small-pox *excite* a diseased action of them?" Till that difficulty be resolved, I shall content myself with matters of fact, and leave theories and speculations to more ingenious men. I am far from supposing that cow-pox will prove any thing like an infallible remedy against any species of atrophia; but being convinced that it has been of essential service in three cases out of four, I have stated the fact, in order that other practitioners may turn their attention to similar cases when they occur in their practice. It is possible these children might have recovered without vaccination; vaccination; but as no other remedy was employed, and as their amendment was *visible* so soon, I cannot but think that the action of cow-pox wrought some important change in their constitutions.

It is to be lamented, that any prejudices still exist amongst the lower class of people against vaccine inoculation, so as to prevent its becomiug more general. Perhaps nothing short of an act of the legislature will produce the desired effect. I am aware that I am treading on sacred ground. I know too, that the liberty of the subject is a very tender point, and must only be touched with the gentlest hand. But I am of opinion, that an effective act might be passed without the smallest infringement of any constitutional principles. From repeated experiments, (made with this express view) I know that a *little* money will remove *very great* difficulties. Suppose then an act was passed, obliging the overseers to pay two shillings and six-pence to every person who would produce a certificate from the surgeon, of the vaccine inoculation having been performed; I am persuaded prejudices and objections amongst the poor would soon die away. The sum thus paid should be put into the rates, and would be no great burden. Surgeons in general would inoculate gratis, or the salary of the *town doctor* might be made adequate to his additional trouble. A clause might be inserted prohibiting any *professional* man from inoculating with the small-pox, not making it penal for any other person to do so; by this means the liberty of the subject would be preserved, whilst the means of exercising it to the injury of the public would be in a great measure destroyed.*

I am, &c.

*Kidderminster, Feb.* 13, 1804. GEORGE CUSTANCE.

